# Making reservoirs cleaner through a Pattern–Process–Effect–Regulation framework

**DOI:** 10.1093/nsr/nwag331

**Published:** 2026-05-29

**Authors:** Xingcheng Yan, Sijiu Zhang, Qiuwen Chen, Jianyun Zhang

**Affiliations:** Center for Eco-Environment Research, Nanjing Hydraulic Research Institute, China; Shallow Lake Ecosystem Monitoring & Experiment Station of Taihu Basin, Nanjing Hydraulic Research Institute, China; Center for Eco-Environment Research, Nanjing Hydraulic Research Institute, China; Center for Eco-Environment Research, Nanjing Hydraulic Research Institute, China; Shallow Lake Ecosystem Monitoring & Experiment Station of Taihu Basin, Nanjing Hydraulic Research Institute, China; Yangtze Institute for Conservation and Development, Nanjing Hydraulic Research Institute, China; Center for Eco-Environment Research, Nanjing Hydraulic Research Institute, China; Shallow Lake Ecosystem Monitoring & Experiment Station of Taihu Basin, Nanjing Hydraulic Research Institute, China; Yangtze Institute for Conservation and Development, Nanjing Hydraulic Research Institute, China

Reservoirs have become one of the most widespread and rapidly expanding freshwater systems on Earth. Constructed for flood management, irrigation, water supply, and hydropower production, they now impound most large rivers worldwide. As of 2024, more than 35 000 reservoirs with a surface area exceeding 10 km^2^ collectively store ∼7420 km^3^ of water [1]. Hydropower remains the dominant renewable energy source globally, accounting for roughly 14.3% of electricity production [2]. The irrigation water supplied by reservoirs has increased dramatically over the past century, from around 18 km^3^ yr^−1^ at the beginning of the 20th century to about 460 km^3^ yr^−1^ by its end [3]. These figures underscore the central role of reservoirs in water, food, and energy systems, while also revealing the scale at which their unintended eco-environmental consequences can challenge sustainable and low-impact development.

**The multifaceted eco-environmental impacts of reservoirs**. The eco-environmental effects of reservoirs arise from tightly coupled physical, chemical, and biological changes. Physically, dam construction fragments river networks and alters natural flow regimes. Worldwide, only about 37% of rivers longer than 1000 km remain free-flowing along their entire course [4]; in Europe, there is on average 0.74 barrier per river kilometer, and in China, ∼62% of major rivers, accounting for 90% of the nation’s total river discharge, have become fragmented [5,6]. Such intensive dam construction severely reduces longitudinal connectivity and poses serious threats to migratory fish populations [7]. Reservoir operation further reshapes downstream hydrological and thermal conditions, affecting reproduction, feeding, and habitat quality of aquatic organisms and contributing to biodiversity loss [8]. Biogeochemically, dams disrupt the transport and transformation of carbon, phosphorus, nitrogen, and silicon, enhancing nutrient retention and altering downstream material fluxes [9]. Reservoirs are also increasingly recognized as greenhouse gas (GHG) sources, with diffusive fluxes alone estimated at ∼0.8 Pg CO₂ equivalents annually [10]. These effects interact across scales and propagate along the aquatic continuum, making reservoir impacts a coupled system problem rather than a set of isolated environmental issues.

**From Pattern–Process to Pattern–Process–Effect–Regulation.** In 1991, Thornton and colleagues published *Reservoir Limnology: Ecological Perspectives* [11], which established a foundational framework for understanding reservoirs through an ecological standpoint. Building on classical lake limnology, they described the longitudinal zonation of reservoirs (for example, riverine, transitional, and lacustrine zones), highlighted how hydrodynamic conditions drive nutrient cycling and biological succession, and emphasized the importance of spatial structure and the processes shaping it. However, unlike natural lakes, reservoirs are highly regulated systems whose hydrological and hydrodynamic regimes are strongly shaped by dam operations, resulting in flow-through characteristics that differ fundamentally from the relatively stable conditions typical of lakes. Nevertheless, the *Pattern–Process* perspective provided a conceptual basis for interpreting spatial patterns and ecological processes in reservoirs, profoundly influencing subsequent reservoir ecology research.

Over the past three decades, reservoir ecology has expanded from explaining spatial heterogeneity and internal biogeochemical processes to predicting ecological outcomes and designing interventions. Issues such as harmful algal blooms, hypolimnetic anoxia, GHG emissions, biodiversity loss, and downstream habitat degradation are not only scientific phenomena to be explained, but also management problems to be anticipated, attributed, and regulated [7–9]. This shift exposes a limitation of the classical Pattern–Process perspective: it is powerful for linking spatial structures to ecological mechanisms, but less explicit in connecting these mechanisms to measurable outcomes, operational choices, and management feedbacks [12].

**Figure 1.**
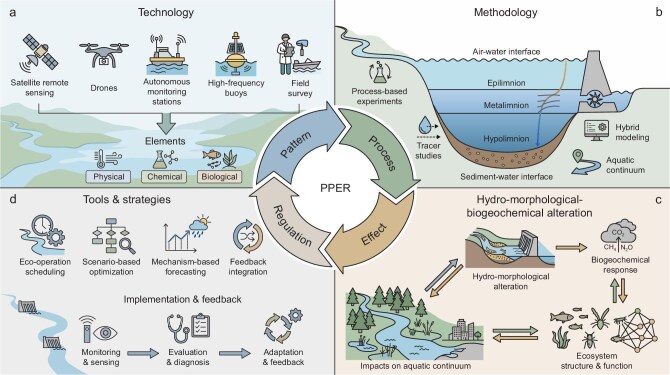
The Pattern–Process–Effect–Regulation conceptual framework for cleaner reservoirs. The framework links four coupled components: (a) pattern, the spatial and temporal organization of physical, chemical, and biological variables; (b) process, the hydrodynamic, biogeochemical, and ecological mechanisms that generate or transform these patterns; (c) effect, the attributable ecological, climatic, and social-ecological outcomes; and (d) regulation, the operational and management interventions designed to alter key processes and reduce undesirable effects. The arrows emphasize that PPER is not a linear extension of the classical Pattern–Process view. Regulation feeds back to reshape reservoir patterns and processes by modifying residence time, stratification, withdrawal depth, flow connectivity, sediment–water exchange, and downstream flow signals, while monitoring of effects updates future regulation. The framework aims to connect diagnosis, mechanism, outcome attribution and adaptive intervention for more sustainable reservoir management.

Addressing these challenges requires a broader view of what constitutes a ‘cleaner’ reservoir, encompassing not only water-quality improvement but also lower GHG emissions, enhanced biodiversity, restored connectivity, improved ecosystem services and reduced downstream impacts. We therefore propose the Pattern–Process–Effect–Regulation (*PPER*) framework as a closed-loop framework rather than a simple extension of the classical view (Fig. 1). In *PPER*, Pattern identifies the spatial and temporal organization of key variables, Process explains the mechanisms that generate or transform these patterns, Effect evaluates attributable ecological, climatic, and social-ecological outcomes, and Regulation represents interventions that feed back to reshape patterns and processes. Compared with driver-pressure-state-impact-response (DPSIR), adaptive management and socio-ecological systems approaches, *PPER* is more reservoir-specific and process-explicit: it links observed patterns, underlying mechanisms, attributable effects, and operational levers within a single feedback logic.

**Operationalizing *PPER* through causal pathways.**  *PPER* becomes operational when the four components are connected through explicit causal pathways. Here, Processes refer to mechanisms that transform reservoir conditions, whereas Effects refer to attributable ecological or climatic outcomes evaluated against baselines or counterfactuals. For example, in a stratified reservoir, persistent thermal stratification may drive hypolimnetic oxygen depletion and nutrient or methane release, increasing algal bloom risk or methane accumulation. Regulation such as selective withdrawal, artificial mixing, hypolimnetic oxygenation, or residence-time control can then be evaluated against counterfactual operating scenarios.

The ecological operation of the Three Gorges Dam provides a retrospective illustration of the same logic [13]. Ecological releases create rising-flow conditions for fish spawning; altered downstream flow patterns affect hydraulic cues, egg drift, and larval transport; and effects can be assessed through spawning scale, egg and larval abundance, recruitment success, and population structure. The operation must also balance flood control, hydropower, navigation, water supply, and local livelihoods. This example illustrates how mechanistic understanding and monitoring feedback can inform adaptive regulation, without implying that the practice was originally designed under *PPER*.

**Advancing reservoir science within the PPER framework.** Looking to the future, advancing reservoir ecology within the *PPER* framework requires coordinated efforts across the four dimensions, while extending the analytical scope toward the aquatic continuum.

- *Pattern: observing reservoirs as engineered–ecological systems.* Understanding spatial patterns is the first step toward predicting ecological consequences. Future research should establish multi-scale observation networks that combine *in situ* high-frequency buoys, autonomous monitoring stations, drones, and satellite remote sensing with sediment and biological surveys [14]. Vertical sensors can capture temperature, dissolved oxygen, pH, conductivity, and related variables across depths, while remote sensing provides spatial information on surface water conditions, such as water color, chlorophyll-a, and algal bloom dynamics. Standardized calibration and quality control are essential for linking observed patterns to underlying processes and for ensuring reproducibility.
- *Process: resolving interfaces that control fluxes and feedbacks.* Building on the spatial patterns, elucidating the mechanisms that operate at key interfaces is essential to understand material and energy fluxes. Research should focus on critical zones, such as the air–water interface, thermal stratification layers, sediment–water interface, and other key boundaries within the reservoir and broader aquatic continuum. Scaled model experiments, tracer studies, and hybrid numerical modeling that combine mechanistic insights with data-driven approaches are essential for improving predictions of nonlinear dynamics and identifying threshold behaviours that drive ecosystem shifts.
- *Effect: quantifying ecological and climate outcomes.* Quantifying effects requires comparable indicators for both beneficial and adverse outcomes of reservoir operation [15]. These should include GHG fluxes, algal bloom risk, biodiversity shifts, fish recruitment, downstream habitat changes, and ecosystem-service trade-offs, with particular attention to how these effects accumulate and propagate along time and space.
- *Regulation: translating process understanding into adaptive operation and governance.* Building on the causal pathways illustrated above, regulation in *PPER* means more than applying existing operation rules; it refers to process-targeted interventions that can be tested, adjusted, and evaluated against ecological outcomes. Once a Pattern–Process–Effect pathway is identified, Regulation should determine which operational lever, such as release timing and magnitude, withdrawal depth, residence-time control, artificial mixing, hypolimnetic oxygenation, sediment management, or cascade coordination, can modify the responsible process, under what constraints, and with what ecological consequences. Because ecological regulation interacts with flood control, hydropower generation, navigation, irrigation, water supply, and local livelihoods, *PPER* also requires consideration of trade-offs, institutional rules, stakeholder coordination, community needs, and cross-reservoir or cross-basin decision contexts. Thus, it complements existing operation and optimization models by linking ecological mechanisms, feasible interventions, and monitoring-based feedback.

**Toward reservoirs as adaptive clean energy infrastructures.** To make *PPER* actionable, future research should focus on four testable tasks: identifying risk-sensitive patterns, resolving controllable processes, quantifying attributable effects, and testing regulation options against explicit counterfactuals. Key questions include which physical, chemical, and biological patterns provide early warning of ecological risks; which processes, such as stratification, oxygen depletion, sediment-water exchange, GHG production, or altered flow cues, can be modified by operation; how these processes translate into water-quality, climate, biodiversity, and downstream effects; and how alternative operating rules perform under ecological, engineering, and socioeconomic constraints. Future applications must also account for long-term ecological effects under climate change, including altered inflow regimes, thermal structure, hypoxia risk, GHG fluxes, and ecological-flow windows. They should also be reservoir-type specific, because large hydropower reservoirs, drinking-water reservoirs, irrigation reservoirs, flood-control reservoirs, and small multipurpose reservoirs differ in dominant processes, feasible interventions, and social constraints. By linking risk diagnosis, mechanistic understanding, outcome attribution, and adaptive regulation, *PPER* can help reservoirs evolve from conventional hydraulic infrastructures toward adaptive, lower-impact engineered–ecological systems.
